# The burden of vision loss in the Middle East and North Africa region, 1990–2019

**DOI:** 10.1186/s13690-023-01188-y

**Published:** 2023-09-26

**Authors:** Erfan Bahremani, Mahasti Alizadeh, Seyed Aria Nejadghaderi, Maryam Noori, Mark J M Sullman, Ali-Asghar Kolahi, Saeid Safiri

**Affiliations:** 1 Social Determinants of Health Research Center, Department of Community Medicine, Faculty of Medicine, Tabriz University of Medical Sciences, Tabriz, Iran; 2 Department of Ophthalmology, Faculty of Medicine, Tabriz University of Medical Sciences, Tabriz, Iran; 3 Neurosciences Research Center, Aging Research Institute, Tabriz University of Medical Sciences, Tabriz, Iran; 4 Systematic Review and Meta-Analysis Expert Group (SRMEG), Universal Scientific Education and Research Network (USERN), Tehran, Iran; 5 Student Research Committee, School of Medicine, Iran University of Medical Sciences, Tehran, Iran; 6 Department of Life and Health Sciences, University of Nicosia, Nicosia, Cyprus; 7 Department of Social Sciences, University of Nicosia, Nicosia, Cyprus; 8 Social Determinants of Health Research Center, Shahid Beheshti University of Medical Sciences, Tehran, Iran; 9 Clinical Research Development Unit of Tabriz Valiasr Hospital, Tabriz University of Medical Sciences, Tabriz, Iran

**Keywords:** Vision loss, Prevalence, Year lived with disability, Global burden of Disease, Middle East and North Africa

## Abstract

**Background:**

The loss of vision is a substantial public health concern that has important implications for an individual’s quality of life. The primary objective of this research was to document the burden of vision loss in the Middle East and North Africa (MENA) region, spanning the years 1990–2019, by age group, sex, underlying cause and sociodemographic index (SDI).

**Methods:**

Publicly available data concerning the burden of vision loss were acquired from the Global Burden of Disease study 2019. The data encompassed all 21 countries within the MENA region for the period spanning 1990 to 2019. The estimates were reported as raw counts and age-standardised rates per 100,000, accompanied by their corresponding 95% uncertainty intervals (UIs).

**Results:**

In 2019, MENA had an age-standardised point prevalence of 7040.0 (95% UI: 6195.0, 8002.7) and an YLD rate of 314.5 (222.1, 427.6) per 100,000 for vision loss, which were 11.1% (-12.5, -9.7) and 24.3% (-27.6, -20.8) lower, respectively, than in 1990. In 2019, Afghanistan [469.6 (333.0, 632.8)] had the largest age-standardised YLD rate and Turkey [210.7 (145.3, 290.9)] had the lowest. All countries showed a decrease in the age-standardised point prevalence and YLD rate between 1990 and 2019, except for Oman, Afghanistan, and Yemen. Furthermore, in 2019 the largest number of prevalent cases and YLDs were found in the 65–69 age group. Also in 2019, the age-standardised YLD rates in MENA exceeded the global averages for most age groups, for both males and females. In 2019, refractive disorders were the most common types of vision loss among children, adolescents, and middle-age adults in MENA, while near vision loss and cataracts were the most common among older adults. Finally, the burden of vision loss had a slightly negatively association with SDI over the period 1990–2019.

**Conclusion:**

Although the burden of vision loss has decreased over the last three decades, the prevalence remains high. These results underscore the importance of healthcare policymakers taking action to implement preventive measures, especially among the elderly and those living in low socioeconomic countries, to decrease the attributable burden in MENA.

**Supplementary Information:**

The online version contains supplementary material available at 10.1186/s13690-023-01188-y.

**Table:** 

**Text box 1. Contributions to the literature**
• There is limited evidence on the morbidity due to vision loss in the Middle East and North Africa (MENA) region over a 30-year period.
• The study identifies significant geographical disparities within MENA, with Afghanistan exhibiting the highest years lived with disability (YLD) rate and Turkey the lowest in 2019.
• The research identifies the 65–69 age group as having the largest number of prevalent cases and YLDs in 2019.

## Introduction

Vision loss and vision impairment are conditions that are associated with a low quality of life, and can increase the risk of depressive symptoms as well as reducing the level of social participation [1, 2]. Moreover, the risk of all-cause mortality is about 1.3 times higher among those with low visual acuity and the risk increases with more severe visual impairment [3]. In 2018, the annual cost of lost productivity from moderate to severe vision impairment and blindness in the Middle East and North Africa (MENA) region was estimated as being $33.6 billion, or 0.35% of gross domestic product [4].

In 2020, the age-standardised point prevalence of blindness was higher in the MENA region than the global level (7.0 vs. 5.3 per 1000 population), although the MENA region also witnessed a larger decrease (than globally) in this rate over the period 1990–2020 (41.5% vs. 27.0% decrease) [5]. However, previous research has estimated that the number of individuals with vision loss will steadily increase up to 2050, due mainly to population growth and aging [5, 6]. Nevertheless, about 76% of the cases across the world have a preventable or treatable cause [7].

Blindness is considerably more prevalent in developing nations, with rates ranging from 10 to 40 times higher than those observed in developed countries [8]. Furthermore, as the majority of the countries in MENA are low- and middle-income countries, understanding the impact of vision loss has great relevance for the region [8]. The VISION 2020 study reported the prevalence and causes of blindness, as well as vision impairment, from 1990 to 2020 [5]. In addition, the prevalence of vision loss attributable to several risk factors or associated conditions has also been reported, including vitamin A deficiency [9], diabetes [10], glaucoma [11], refraction disorders [12], cataract [13] and other causes [14]. Moreover, using Global Burden of Disease (GBD) 2015 data several studies have reported the burden and prevalence of vision loss in the Eastern Mediterranean Region (EMR) [15, 16]. However, there has been no previous research on the burden of vision loss specifically on the MENA region. Furthermore, the information from earlier studies is now outdated and more up-to-date information is needed. Thus, we reported the burden of vision loss in the MENA region from 1990 to 2019 by sex, age group, underlying cause and sociodemographic index (SDI) using data obtained from GBD 2019.

## Methods

### Overview

The GBD 2019 project has compiled data on 369 diseases and injuries as well as 87 risk factors across 204 nations for the time frame spanning 1990–2019 [17, 18]. Although vision loss is a common health problem its burden has not yet been reported for all regions of the world. With this in mind, the current research utilised GBD 2019 data to identify the burden of vision loss for all nations in the MENA region over the 1990–2019 period. The MENA region contains 21 countries, which are: Afghanistan, Algeria, Bahrain, Egypt, Iran, Iraq, Jordan, Kuwait, Lebanon, Libya, Morocco, Oman, Palestine, Qatar, Saudi Arabia, Sudan, the Syrian Arab Republic, Tunisia, Turkey, the United Arab Emirates and Yemen. An in depth description of the GBD 2019 methodology used to model the burden attributable to vision loss is available in the GBD capstone papers [17, 18] and all data can be viewed using these hyperlinks: https://vizhub.healthdata.org/gbd-compare/ and http://ghdx.healthdata.org/gbd-results-tool.

### Case definition and data sources

The case definition used here was having a visual acuity score of less than 6/18 on the Snellen chart. The vision loss data was obtained from surveys that measured visual acuity in population representative samples. The survey data came from either record data, peer-reviewed publications or the grey literature. Surveys were omitted if that did not provide data that could be transformed into the Snellen scale, or did not measure “presenting” or “best-corrected” vision. Presenting vision is the visual acuity measured while the patients are wearing their current glasses. In contrast, regardless of the strength of glasses used by the patients, the best corrected vision is the best available correction for their refractive error. Previous research has reported the prevalence of vision loss that was stratified according to the cause, and this information was utilised to calculate the prevalence of vision loss attributable to each cause (e.g., diabetic retinopathy, glaucoma, cataracts, macular degeneration, and other causes).

A systematic review was undertaken during GBD 2015, which identified sources that had been published since the previous systematic review in GBD 2013. Moreover, information from large surveys that were nationally representative, such as the WHO Study on Global Ageing and Adult Health (SAGE) and the National Health and Examination Surveys (NHANES) from the United States, were also incorporated. Furthermore, the Rapid Assessment of Avoidable Blindness (RAAB) repository, which is a database containing research on vision loss studies that have been conducted in developing countries across the world, was also used to provide data for GBD 2016 and GBD 2017. In contrast, the data extraction for GBD 2019 was undertaken by GBD collaborators from the Vision Loss Expert Group (VLEG). Following an exhaustive systematic literature review, all abstracts that passed screening were then referred to regional expert groups to evaluate their quality and to decide whether they should be included. The search covered the time period 1980–2018 and included the following databases: MEDLINE, Embase, WHOLIS, SciELO, Open Grey and a grey literature search that was commissioned by VLEG (York Health Economics Consortium, UK).

### Data processing and disease model

The modelling of vision loss was undertaken in two separate stages. The first stage involved estimating the overall prevalence of presenting vision loss, which included: moderate vision loss, severe vision loss, blindness, and near vision loss (presbyopia). The prevalence of presbyopia was obtained directly at this stage. The modelling of the three remaining types (moderate vision loss, severe vision loss, blindness) continued at the second stage.

#### Estimate severity-specific vision loss (the “envelopes”)

DisMod-MR 2.1 was utilised to calculate the overall prevalence figures for the different types of vision impairment, which were moderate vision loss, severe vision loss, blindness, near vision loss, and the combined category of presenting vision loss (which includes moderate, severe, and blindness). To ensure consistency among the different levels of severity, the presenting vision loss model was also included as a covariate in the severity-specific models. A meta-regression (MR-BRT) with a cubic spline on age was used to separate the severity data. In addition, the healthcare access and quality index (HAQI) and socio-demographic Index (SDI) were used as location specific covariates, which were used as proxy measures for access to eye care.

#### Estimated cause-specific vision loss

The second stage involved modelling vision loss by cause: cataract, diabetic retinopathy, encephalitis, glaucoma, macular degeneration, meningitis, onchocerciasis, retinopathy of prematurity, trachoma, uncorrected refractive error, vitamin A deficiency, and a residual category of other types of vision loss. The attributable burden of vision loss due to diabetic retinopathy, encephalitis, meningitis, onchocerciasis retinopathy of prematurity, and vitamin A deficiency were estimated as part of their underlying cause. Two DisMod-MR 2.1 models were run each for cataracts, glaucoma, macular degeneration, and other vision loss, one that combined the moderate and severe categories of vision loss, and another for blindness. The reason for combining the two types of vision loss was that they were mostly available combined. Refractive error was estimated using three different models, one for each level of severity. The combined moderate plus severe vision loss estimates were split into moderate and severe for each cause using the ratio of moderate and severe vision loss envelopes. For each severity level, the cause-specific prevalence of vision loss was fitted to the total prevalence of the vision loss envelopes. This process produced the prevalence of vision loss of each severity level that were attributable to each cause.

### Years lived with disability

Table S1 presents the severity levels, disability weights (DWs) and lay descriptions of vision loss. The DWs were sourced from the GBD disability weight survey [17]. The years of life lost as a result of premature mortality and the years lived with disability (YLDs) were combined to form disability-adjusted life year (DALY), which is a frequently used statistic for indicating the burden of a disease [17]. As there was no mortality due to vision loss, the DALY and YLD estimates were the same [17]. The DWs were multiplied with the prevalence estimates of each of the four severity level to estimate the YLDs due to vision loss. Finally, 1000iterations were undertaken at each step, which were combined with uncertainty from residual non-sampling error, input data, and measurement error. The 95% uncertainty intervals (UIs) consisted of the 25th and 975th values of the numerically ordered iterations and accompanied all estimates.

### Compilation of results

Smoothing splines were utilised to investigate the relationship the YLDs attributable to vision loss had with the SDIs for all MENA countries [19]. The SDI metric, which ranges from 0 (indicating the least developed) to 1 (representing the most developed) incorporates three components: (1) gross domestic product per capita smoothed over the previous 10 years; (2) mean number of years of schooling for those aged 15 years and older; and (3) total fertility rate in those less than 25 years old. R software (V. 3.5.2) was used to perform all analyses and create all figures.

## Results

### The middle east and north africa region

In 2019, the MENA region had a total of 32.5 million (95% UI: 28.6 to 36.6) cases and an age-standardised point prevalence of vision loss of 7040.0 (ranging from 6195.0 to 8002.7) per 100,000. This prevalence rate was notably 11.1% (-12.5 to -9.7) lower than the rate observed in 1990 (Table 1 and Table S2). Furthermore, in 2019, the region recorded 1.4 million (1.0 to 1.9) YLDs attributable to vision loss and an age-standardised rate of 314.5 (222.1 to 427.6) YLDs per 100,000 population, which has decreased by 24.3% (-27.6 to -20.8) since 1990 (Table 1 and Table S3).

**Table 1:** Prevalent cases and YLDs due to vision loss in 2019 and the percentage change in the age-standardised rates during the period 1990–2019

	Prevalence (95% UI)			YLDs (95% UI)		
	Counts (2019)	ASRs (2019)	Pcs in ASRs 1990–2019	Counts (2019)	ASRs (2019)	Pcs in ASRs 1990–2019
North Africa and Middle East	32,493,045 (28,615,366, 36,578,053)	7040 (6195, 8002.7)	-11.1 (-12.5, -9.7)	1,392,915 (974,014, 1,910,579)	314.5 (222.1, 427.6)	-24.3 (-27.6, -20.8)
Afghanistan	1,474,739 (1,314,180, 1,645,083)	8669.4 (7701.8, 9803.8)	-3.6 (-7.7, 0.4)	70,486 (49,969, 97,331)	469.6 (333, 632.8)	-3.2 (-8.2, 2.4)
Algeria	2,511,456 (2,197,306, 2,862,411)	7245 (6385.7, 8220.5)	-10.5 (-13.8, -6.5)	107,179 (75,493, 146,663)	321.4 (227.7, 436.8)	-24.1 (-27.6, -20)
Bahrain	76,991 (66,529, 89,429)	6935.5 (6090.8, 7906.9)	-10.5 (-14, -7.4)	2871 (1974, 4061)	286.9 (201.3, 392.2)	-25.3 (-29.5, -20.9)
Egypt	5,286,491 (4,663,456, 5,958,968)	7587.6 (6689.1, 8589)	-11 (-14.9, -7.6)	216,390 (148,682, 300,116)	330.5 (230.6, 453.4)	-25.1 (-28.9, -21)
Iran	5,189,421 (4,674,982, 5,752,587)	7008.8 (6299.5, 7778)	-13.7 (-14.7, -12.8)	270,459 (191,734, 365,108)	376.1 (267.1, 505.8)	-24.9 (-27.2, -22.2)
Iraq	1,894,229 (1,668,248, 2,145,094)	7188.6 (6332, 8194.2)	-11.8 (-15.5, -8)	78,123 (53,982, 108,201)	316.4 (224, 429.6)	-23.9 (-27.6, -20)
Jordan	466,915 (405,676, 535,643)	6310.5 (5472.6, 7295.9)	-10.8 (-14.8, -7.2)	16,879 (11,445, 23,944)	240.7 (167.7, 334.3)	-22.7 (-26.8, -18.5)
Kuwait	201,027 (175,623, 228,226)	6651.8 (5863.9, 7604.1)	-8.6 (-12.2, -4.9)	7646 (5182, 10,814)	271.7 (191.1, 373.8)	-19.1 (-22.4, -15.5)
Lebanon	385,543 (342,104, 435,490)	7436.8 (6593, 8386.3)	-11.8 (-15.8, -7.7)	15,959 (11,170, 21,721)	307.7 (215.6, 419.2)	-25.9 (-30.2, -20.9)
Libya	376,092 (329,144, 427,264)	7050.9 (6199.1, 8055.7)	-10.1 (-13.7, -6.7)	16,762 (11,915, 22,769)	330.2 (233.9, 448.1)	-22 (-26.9, -16.8)
Morocco	2,184,191 (1,873,215, 2,523,626)	6959.9 (6023.5, 8005.7)	-7.8 (-11.9, -3.1)	83,195 (57,658, 116,064)	275.4 (193, 379.9)	-18.4 (-23, -13.7)
Oman	241,576 (214,912, 267,605)	9139 (8275.2, 10048.7)	5.4 (-0.6, 12.5)	10,623 (7367, 14,582)	455 (325.7, 615.3)	1.7 (-6, 9.7)
Palestine	204,793 (180,608, 231,746)	7198.3 (6302.3, 8226.7)	-13.3 (-16.7, -9.3)	7957 (5452, 11,096)	299.4 (210.7, 410.3)	-26.4 (-30.2, -22.6)
Qatar	102,405 (88,628, 117,818)	6790.4 (5968.2, 7648.3)	-12.8 (-16.2, -9.3)	3840 (2602, 5445)	287.4 (202.4, 394.4)	-29.9 (-35.2, -24.4)
Saudi Arabia	1,824,520 (1,641,659, 2,026,299)	8343.9 (7539.1, 9197.3)	-20.9 (-23.8, -18)	89,963 (63,397, 122,806)	464.8 (332, 621.9)	-42.2 (-46.1, -37.8)
Sudan	1,660,823 (1,463,490, 1,863,433)	7423.2 (6473.5, 8474.1)	-13 (-16.6, -9.5)	77,359 (54,679, 106,068)	373.4 (266.5, 503.1)	-26.8 (-31.4, -21.3)
Syrian Arab Republic	929,351 (811,162, 1,053,224)	7392.1 (6532.1, 8363)	-11.2 (-14.3, -7.7)	39,642 (27,816, 54,424)	331.2 (233.8, 451)	-25.6 (-29.3, -21.3)
Tunisia	791,531 (673,946, 927,901)	6454.9 (5518.5, 7537.4)	-11.9 (-15.7, -7.9)	33,503 (23,412, 45,643)	278.7 (196.6, 378)	-29.9 (-34.4, -25)
Turkey	5,118,215 (4,339,121, 5,961,223)	5923.4 (5060.1, 6903.4)	-8.9 (-14, -3.7)	179,407 (123,563, 248,271)	210.7 (145.3, 290.9)	-23.2 (-28.3, -17.7)
United Arab Emirates	356,229 (301,396, 412,926)	6690.6 (5880.4, 7633.2)	-12 (-15.1, -8.8)	13,430 (9159, 18,934)	288.5 (202.2, 392.1)	-24.1 (-27.5, -20.5)
Yemen	1,183,493 (1,033,235, 1,344,694)	7385.7 (6405.5, 8500.9)	-1.1 (-6.6, 4.6)	49,827 (34,834, 68,627)	335.5 (238, 452.9)	-6.2 (-12, 0.3)

### Country level

**Fig. 1:**
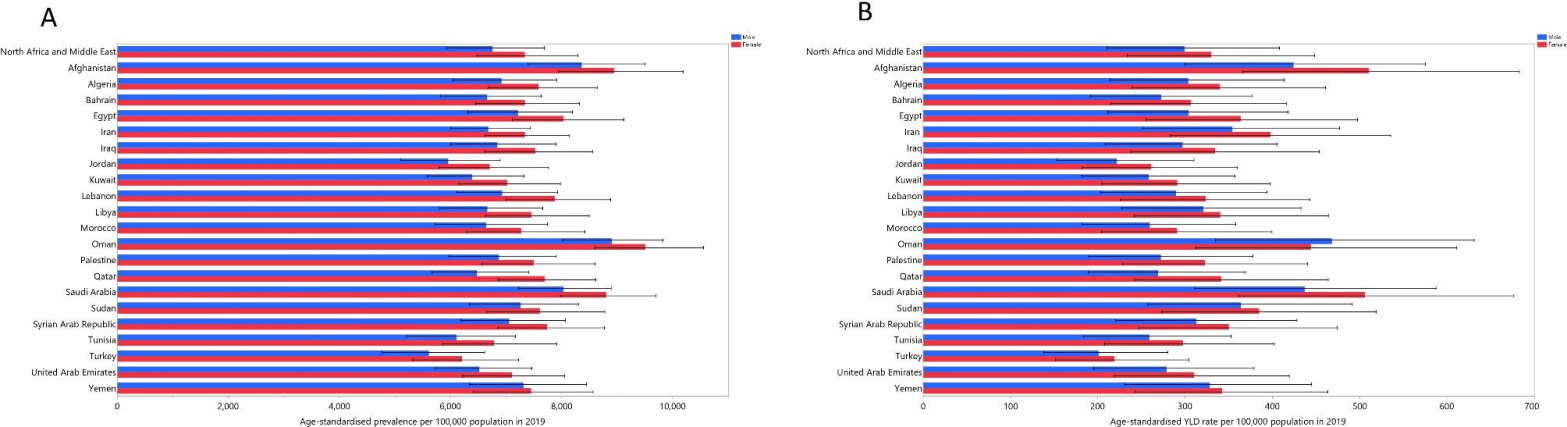
Age-standardised point prevalence (**A**) and YLD rate (**B**) of vision loss (per 100,000 population) in the Middle East and North Africa region in 2019, by sex and country. YLD = year lived with disability. (Generated from data available from http://ghdx.healthdata.org/gbd-results-tool)

The estimated age-standardised point prevalence of vision loss in 2019 varied across the countries located in MENA, varying from 5923.4 to 9139.0 cases per 100,000. In 2019, Oman [9139.0 (8275.2 to 10048.7), Afghanistan [8669.4 (7701.8 to 9803.8)] and Saudi Arabia [8343.9 (7539.1 to 9197.3)] recorded the highest age-standardised point prevalence rates, while Turkey [5923.4 (5060.1 to 6903.4)], Jordan [6310.5 (5472.6 to 7295.9)] and Tunisia [6454.9 (5518.5 to 7537.4)] were lowest (Table 1 and Table S2). The age-standardised point prevalence of vision loss is presented in Fig. 1A by country and sex for 2019.

The national age-standardised YLD rate of vision loss varied from 210.7 to 469.6 cases (per 100,000) in 2019. Afghanistan [469.6 (333.0 to 632.8)], Saudi Arabia [464.8 (332.0 to 621.9)] and Oman [455.0 (325.7 to 615.3)] had the largest rates, while the lowest were found in Turkey [210.7 (145.3 to 290.9)], Jordan [240.7 (167.7 to 334.3)] and Kuwait [271.7 (191.1 to 373.8)] (Table 1 and Table S3). The age-standardised YLD rates of vision loss in 2019 are reported by country and by sex in Fig. 1B.

Significant decreases in the age-standardised point prevalence were seen in eighteen of the countries over the measurement period, with the exceptions being Oman [5.4% (-0.6 to 12.5)], Yemen [-1.1% (-6.6 to 4.6)] and Afghanistan [-3.6% (-7.7 to 0.4)]. The largest decreases were found in Saudi Arabia [-20.9% (-23.8 to -18.0)], Iran [-13.7% (-14.7 to -12.8)] and Palestine [-13.3% (-16.7 to -9.3)] (Figure S1 and Table S2). Moreover, all countries had decreases in the age-standardised YLD rates, except for Oman [1.7% (-6.0 to 9.7)], Afghanistan [-3.2% (-8.2 to 2.4)] and Yemen [-6.2% (-12.0 to 0.3)]. The largest decreases were found in Saudi Arabia [-42.2% (-46.1 to -37.8)], Qatar [-29.9% (-35.2 to -24.4)] and Tunisia [-29.9% (-34.4 to -25.0)] (Figure S2 and Table S3).

### Relationship with age and sex

**Fig. 2:**
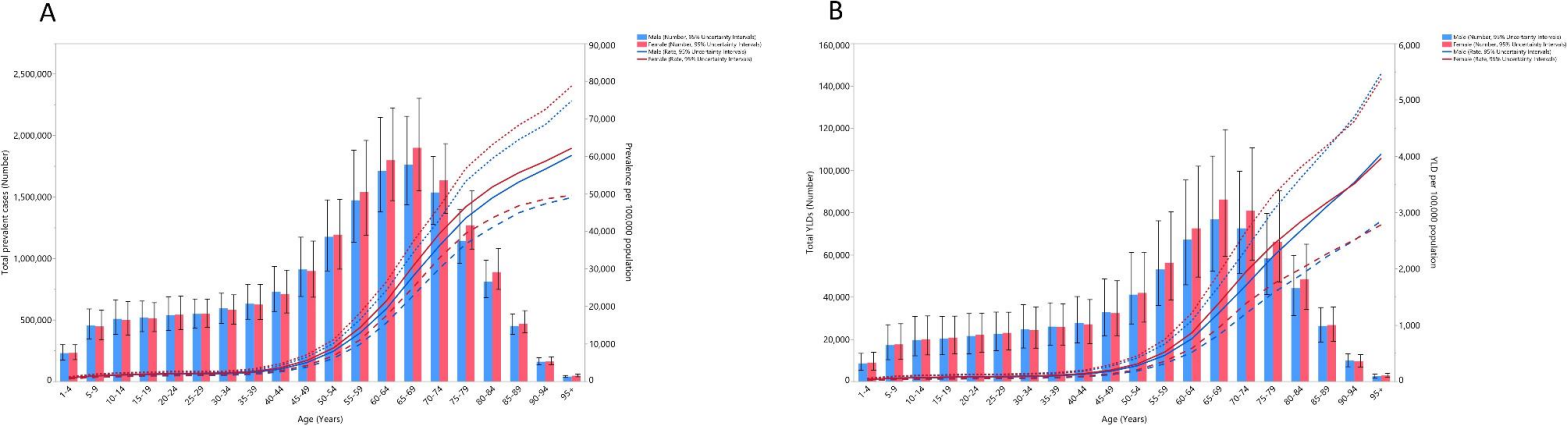
Numbers of prevalent cases and point prevalence (**A**) and the number of YLDs and YLD rate (**B**) for vision loss per 100,000 population in the Middle East and North Africa region, by age and sex in 2019; Dotted and dashed lines indicate 95% upper and lower uncertainty intervals, respectively. YLD = year lived with disability. (Generated from data available from http://ghdx.healthdata.org/gbd-results-tool)

In 2019, the prevalence of vision loss in the MENA region rose with increasing age and reached their highest level in the 65–69 age group for both females and males, before decreasing with age. Point prevalence also increased with aging for males and females, but the age-standardised point prevalence had no significant sex differences (Fig. 2A). Similarly, the YLDs peaked in the 65–69 age range for both sexes, followed by a decline with increasing age. In addition, the age-standardised YLD rate became larger with age. The YLDs and the YLD rate did not significant differ between males and females (Fig. 2B).

**Fig. 3:**
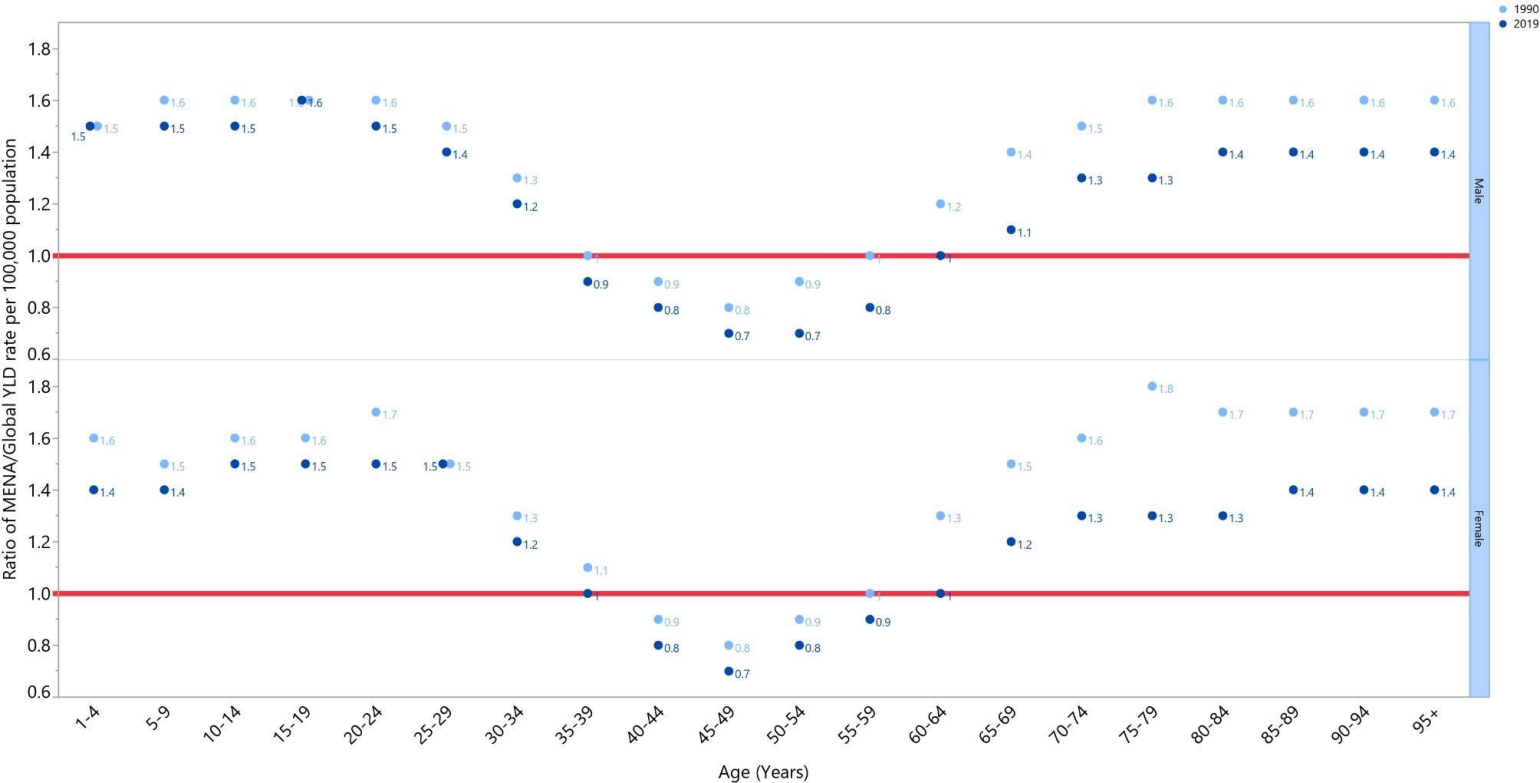
Ratio of the Middle East and North Africa region to the global YLD rate by age and sex, 1990 and 2019. YLD = year lived with disability. (Generated from data available from http://ghdx.healthdata.org/gbd-results-tool)

In MENA, the vision loss-associated with the YLD rate in 2019 were higher than the global YLD rate for all age groups except for the 35–64 age range in both sexes. Furthermore, in comparison to the global rate, the highest YLD rates in 2019 were found among 15–19 year old males (1.6) and in 10–29 year old females (1.5) (Fig. 3).

### Underlying cause

**Fig. 4:**
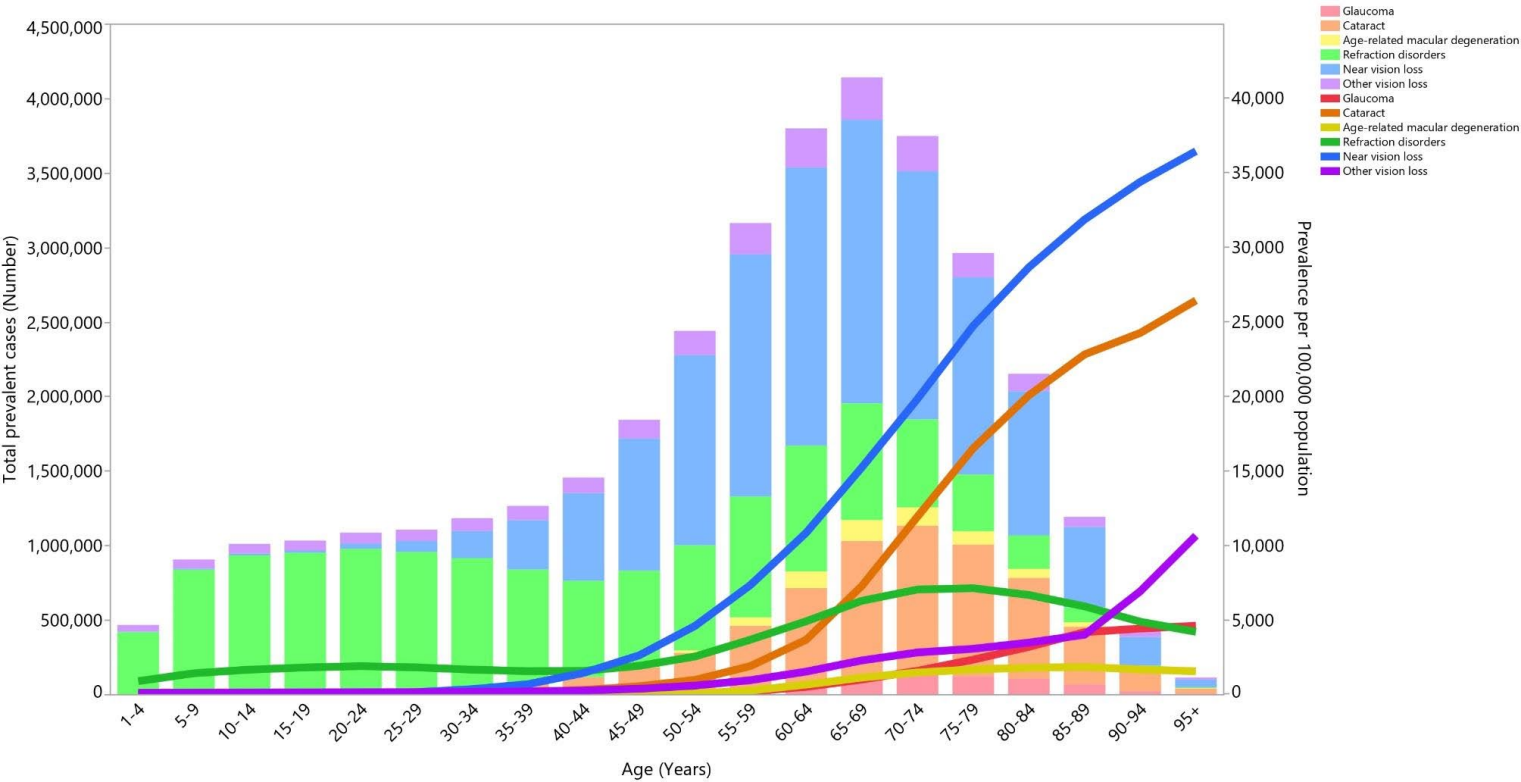
Numbers of prevalent cases and point prevalence per 100,000 population attributable to each underlying cause of vision loss in the Middle East and North Africa region by age in 2019. YLD = year lived with disability. (Generated from data available from http://ghdx.healthdata.org/gbd-results-tool)

In 2019, refractive disorders exhibited the highest prevalent and point prevalence up to 44 years of age. In comparison, among those aged 45 and above near vision loss displayed the highest point prevalence, with refractive disorders and cataracts second and third highest (Fig. 4).

### Relationship with the socio-demographic index (SDI)

**Fig. 5:**
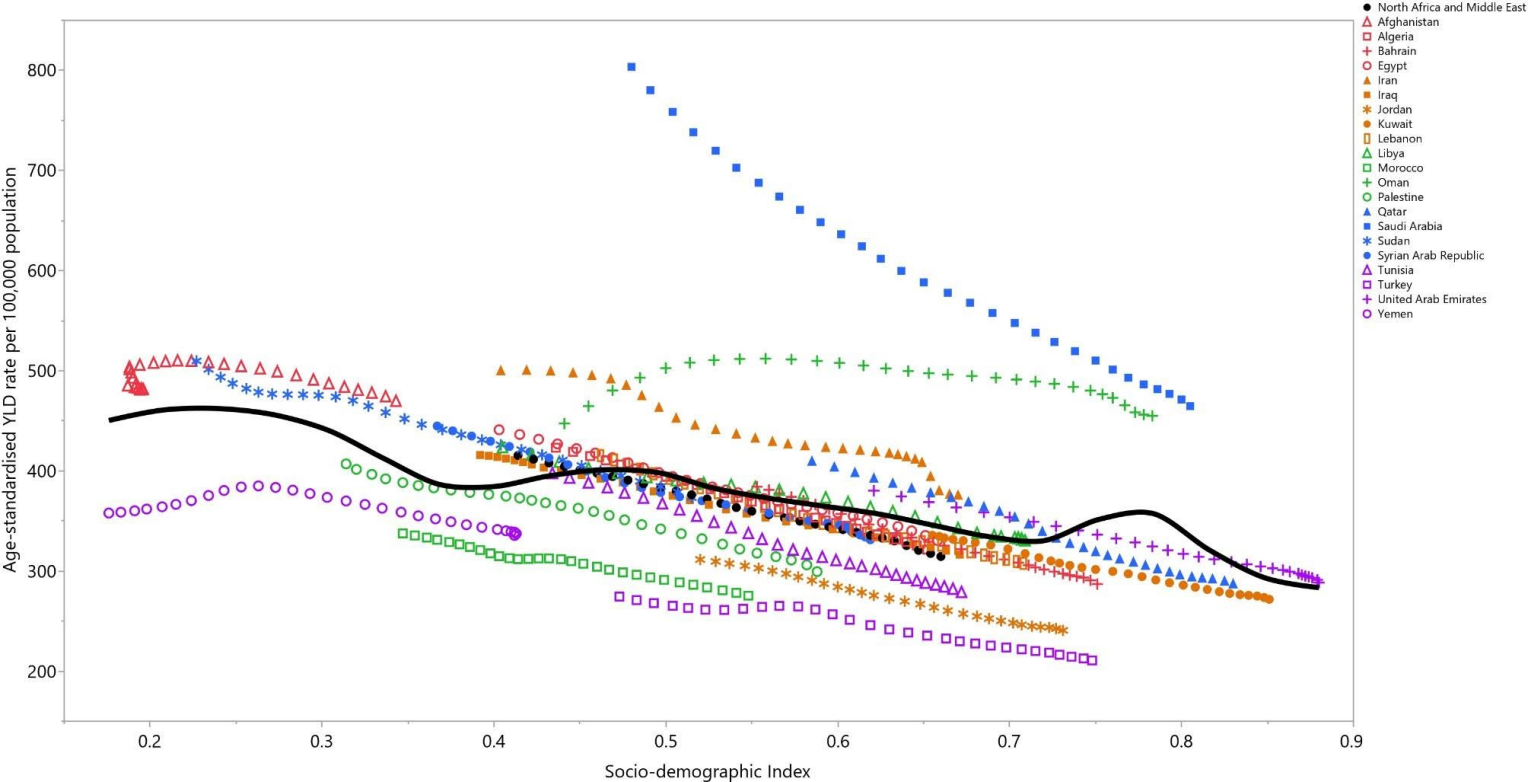
Age-standardised YLD rates of vision loss for 21 countries and territories by SDI, 1990–2019; Expected values based on the Socio-demographic Index and disease rates in all locations are shown as the black line. Each point shows the observed age-standardised YLD rate for each country during 1990–2019. YLD = year lived with disability. SDI = Socio-demographic Index (Generated from data available from http://ghdx.healthdata.org/gbd-results-tool)

The regional YLD rate of vision loss had a negative correlation with the SDI level from 1990 to 2019. Saudi Arabia, Iran, Oman and Afghanistan had higher than the expected YLD rates from 1990 to 2019, while Turkey, Yemen, Morocco and Jordan had rates that were lower than expected (based upon their SDI). Iraq, Syria, Tunisia, Qatar, the United Arab Emirates, Egypt, Algeria and Libya reached a lower-than-expected YLD rate over this time period (Fig. 5).

## Discussion

The burden attributable to vision loss in MENA was lower in 2019 than it was in 1990. A decreasing trend in the age-standardised point prevalence and YLD rates of vision loss were observed in almost all countries. The highest attributable burden in 2019 was found among the elderly and those residing in the least developed countries. Refractive disorders, near vision loss (hyperopia) and cataracts were the most common contributors to vision loss among the MENA countries.

We found there was an 11.1% decrease in the age-standardised point prevalence, as well as a 24.3% reduction in the YLD rate associated with vision loss from 1990 to 2019. Moreover, the annual percent change in the age-standardised point prevalence and DALYs attributable to blindness (0.39%) and vision loss (0.95%) decreased significantly in MENA over the period 1990–2019 [14]. Another study that utilised GBD 2019 data also demonstrated that the regional point prevalence and YLD rate of blindness declined by 41.5% and 41.1%, respectively, and the attributable rates of moderate and severe vision impairment decreased by 6.1% and 10.9%, respectively, over the period 1990–2019 [20]. Furthermore, in 2015 the age-standardised prevalence and YLD rate due to vision loss were 15,500 and 482.3 per 100,000 in the EMR, respectively [15]. Our study found an age-standardised point prevalence of 7040 (per 100,000) in 2019 and a YLD rate of vision loss of 314.5 (per 100,000) in MENA. Although a direct comparison with our study would not be reasonable, due to variations in the methodologies used, an overall reduction in the point prevalence and YLD rate, relative to the previously mentioned study, was observed. Improvements in controlling some infectious causes of vision loss, like trachoma and onchocerciasis, and increases in education can partially explain the reduced prevalence of vision loss in the region [21]. Furthermore, we found that the MENA/Global YLD ratio was above 1 for most age groups, among both sexed, and in both 1990 and 2019. In support of our findings, one study in 2015 showed that, in comparison to the rest of the world, the age-standardised point prevalence of blindness (1000 vs. 500 per 100,000), mild vision impairment (3500 vs. 2500 per 100,000) and moderate to severe vision impairment (4600 vs. 2900 per 100,000) were higher in MENA among all age groups and for both sexes [16]. Poor quality services and low population awareness of eye screening in the region and the low availability of medical technologies, particularly in low-income countries in MENA, could lead to the higher prevalence of vision loss and the substantial burden in MENA [21].

Although most MENA countries had declines in the point prevalence and YLD rate during the last three decades, there were no reductions found in Oman, Afghanistan and Yemen. In contrast, previous research reported that the biggest reductions in the point prevalence (26.6%) and YLD rate (16.2%), between 1990 and 2015, were found in Oman [15]. Population aging and the transition from communicable to chronic eye diseases may be one of the reasons that there was no decrease in Oman’s burden from vision loss [22]. Nevertheless, future national and sub-national studies are needed to evaluate the vision loss burden in these three countries. In addition, Iran had one of the largest decreases in the point prevalence of vision loss in the region. Iran’s successful initiatives, which are recommended to other countries, include: (1) improving community education, (2) implementing interventions in the social determinants of health and (3) increasing access to healthcare services [23]. At the global level, females had a higher age-standardised DALY rate of blindness and vision loss in both 1990 (3.11 vs. 2.96 per 1000) and 2019 (2.91 vs. 2.64 per 1000) [14]. In accordance with their findings, Fig. 1A shows that females had a higher age-standardised YLD rate in 20 of the 21 countries studied here, with Oman being the one exception. Previous research in the EMR also observed that females had a higher age-standardised point prevalence than males in 2015 (p < 0.001) [15]. There are several potential factors that might explain the higher prevalence of vision loss among women, including the longer life expectancy in females (which can result in a rise in age-related ocular diseases), lower use of primary healthcare services and biological factors [24].

The current research found the age-standardised point prevalence and YLD rate rose with age. In accordance with our results, one study in 2020 found a positive relationship between the age-specific global point prevalence of impaired distant vision and age [5]. Moreover, globally the DALY rates attributable to different subtypes of blindness and vision loss increased with advancing age in both 2019 and 2020 [5, 14]. There are several genetic, environmental and lifestyle factors among the elderly that can lead to impairment in spatial contrast sensitivity, scotopic contrast sensitivity and light sensitivity and could increase the prevalence of vision loss in this population [25].

Our study showed that in 2019 cataracts, refractive disorders and near vision loss were the three most common causes of vision loss. In line with our finding, a study showed that other vision loss, refractive and accommodation disorders and cataracts accounted for 0.25%, 0.23% and 0.19% of all DALYs (out of 291 conditions) in 2010, and these three accounted for the largest proportion of DALYs among ophthalmologic conditions [26]. In 2015, in the EMR the largest proportion of prevalent cases in all age groups were from refractive and accommodation disorders, followed by cataracts [15]. In 2020, cataracts, other conditions and uncorrected refractive disorders were the leading causes of blindness globally [5, 21]. Their findings are mostly in agreement with our results and the minor differences could be as a result of variations in the reporting of different parameters or changing the codes and names of attributable diseases. Furthermore, in 2019 a study showed that refractive disorders (36.9%) and cataracts (27.0%) contributed the largest proportion of DALYs attributable to blindness and vision loss in MENA [14]. One of the causes of the high prevalence of refractive disorders in the region is the strong inclination of individuals to wear contact lens instead of spectacles [8]. In addition, the high prevalence of vision loss attributable to cataracts could be due to the low number of ophthalmologists, or age and sex disparities in access to cataract surgery [27, 28]. Therefore, an interdisciplinary system is required for the control and prevention of blindness due to this easily treatable condition.

There are three groups that should be involved in preventive programs for vision loss, which are the government, private organisations and the population [8]. There are a number of recommendations to lower the of vision loss burden in MENA, which include: (1) public eye health education regarding childhood vaccinations and the need to manage ocular surface infections early; (2) the continuing medical education of general practitioners for the management of common eye diseases to reduce the overloaded specialty services; (3) development of pathways for screening and monitoring patients with glaucoma, cataracts or underlying conditions such as diabetes; (4) a reduction in the cost to the patient, increased access to eye healthcare services, and providing insurance coverage for all ophthalmology services and treatments; (5) building resource capacity and increasing support for countries with shortages in eye health; and (6) controlling blood pressure, hyperlipidaemia and blood glucose, as well as advocating smoking cessation in primary care [8, 21].

In 2015, research showed that with each 0.1 unit increase in SDI, the age-standardised YLD rate of all causes of vision loss decreased by 23.9% (-26.7 to -21.2) in the EMR [15]. Moreover, at the global level, the relationship between the age-standardised DALYs rates of the different eye diseases and the SDI was broadly negative in 2019 [14]. In accordance with the two above-mentioned articles, we found that the age-standardised YLD rates decreased with increases in the SDI levels. Similarly, a study which investigated socio-economic disparities in the worldwide burden of near vision loss revealed that the age-standardised DALY rate had a negative correlation with the human development index (HDI) (standardized β = −0.68, P < 0.001) [29]. In addition, in 2017 there was an inverse relationship found between the worldwide age-standardised YLD rate of vision loss attributable to vitamin A deficiency and HDI (r = − 0.24, P = 0.01) [9]. A lack of expert ophthalmologists and fully equipped clinics in low-income countries might delay or prevent early diagnosis and treatment of eye disorders, which may result in higher disabilities. This research represents the most recent effort to evaluate the burden attributable to vision loss in the MENA region using modelling approaches. Nevertheless, it is important to acknowledge shortcomings when interpreting the findings that must be taken into consideration when reading the results. First and foremost, as is the case in other GBD studies, data sparsity, especially in low-income countries, can lead to bias in the reporting of results. Also, since we used modelling strategies for countries that had no data on the attributable burden, the data are based on estimates rather than actual data. Secondly, we only reported the burden by age and sex, while other demographic factors, such as the level of education or employment, were not reported. Thirdly, there might be some variations in the definitions and diagnoses of some of the conditions related to vision loss. Fourthly, the measurement of visual acuity would be difficult for children, so the estimates for this population might not be reliable. Fifthly, the burden of vision loss was not reported at the sub-national level or by location of residence (i.e. rural and urban), which is a highly recommended addition for future GBD iterations. Lastly, it would be better to access more accurate data and to employ a more precise definition of vision loss. More specifically, it might be better to use the World Health Organization definition for presenting vision. This can be considered in further GBD iterations. Moreover, it is important to update the burden regularly and to incorporate the effect of the Coronavirus disease 2019 pandemic on vision loss.

## Conclusions

Although the burden attributable to vision loss decreased over the past three decades, vision loss still causes a large number of YLDs in MENA. The findings of our study demonstrate that there is a need to implement programs for the management and control of refractive disorders and cataracts. Moreover, these programs should prioritise the elderly and those residing in low socioeconomic countries. Future research is needed to track the trends in vision loss over the next decades and to offer novel strategies for targeting the attributable burden of the disease more efficiently.

### Electronic supplementary material

Below is the link to the electronic supplementary material.

Additional File 1: Table S1. Sequelae for vision loss and the associated disability weights from the Global Burden of Disease 2019 Study.

Additional File 2: Table S2. Prevalence of vision loss in 1990 and 2019 for both sexes and the percentage change in the age-standardised rates (ASRs) per 100,000 in the North Africa and the Middle East region. (Generated from data available from http://ghdx.healthdata.org/gbd-results-tool).

Additional File 3: Table S3. YLDs due to vision loss in 1990 and 2019 for both sexes and the percentage change in the age-standardised rates (ASRs) per 100,000 in the North Africa and the Middle East region. (Generated from data available from http://ghdx.healthdata.org/gbd-results-tool).

Additional File 4: Figure S1. The percentage change in the age-standardised point prevalence of vision loss in the Middle East and North Africa region from 1990 to 2019, by sex and country. (Generated from data available from http://ghdx.healthdata.org/gbd-results-tool)

Additional File 5: Figure S2. The percentage change in the age-standardised YLD rate of vision loss in the Middle East and North Africa region from 1990 to 2019, by sex and country. (Generated from data available from http://ghdx.healthdata.org/gbd-results-tool)

## Data Availability

The data used for these analyses are all publicly available at http://ghdx.healthdata.org/gbd-results-tool.

## References

[1] Nayeni M, Dang A, Mao AJ, Malvankar-Mehta MS (2021). Quality of life of low vision patients: a systematic review and meta-analysis. Can J Ophthalmol.

[2] Shah K, Frank CR, Ehrlich JR (2020). The association between vision impairment and social participation in community-dwelling adults: a systematic review. Eye.

[3] Ehrlich JR, Ramke J, Macleod D, Burn H, Lee CN, Zhang JH (2021). Association between vision impairment and mortality: a systematic review and meta-analysis. The Lancet Global Health.

[4] Marques AP, Ramke J, Cairns J, Butt T, Zhang JH, Muirhead D (2021). Global economic productivity losses from vision impairment and blindness. EClinicalMedicine.

[5] Bourne R, Steinmetz JD, Flaxman S, Briant PS, Taylor HR, Resnikoff S (2021). Trends in prevalence of blindness and distance and near vision impairment over 30 years: an analysis for the global burden of Disease Study. The Lancet Global Health.

[6] Bourne RRA, Flaxman SR, Braithwaite T, Cicinelli MV, Das A, Jonas JB (2017). Magnitude, temporal trends, and projections of the global prevalence of blindness and distance and near vision impairment: a systematic review and meta-analysis. The Lancet Global Health.

[7] Bourne RRA, Stevens GA, White RA, Smith JL, Flaxman SR, Price H (2013). Causes of vision loss worldwide, 1990–2010: a systematic analysis. The Lancet Global Health.

[8] Tabbara KF (2001). Blindness in the eastern Mediterranean countries. Br J Ophthalmol.

[9] Xu Y, Shan Y, Lin X, Miao Q, Lou L, Wang Y (2021). Global patterns in vision loss burden due to vitamin A deficiency from 1990 to 2017. Public Health Nutr.

[10] Xu Y, Wang A, Lin X, Xu J, Shan Y, Pan X (2021). Global burden and gender disparity of vision loss associated with diabetes retinopathy. Acta Ophthalmol.

[11] Sun Y, Chen A, Zou M, Zhang Y, Jin L, Li Y (2022). Time trends, associations and prevalence of blindness and vision loss due to glaucoma: an analysis of observational data from the global burden of Disease Study 2017. BMJ Open.

[12] Li H-Y, Liu Y-M, Dong L, Zhang R-H, Zhou W-D, Wu H-T (2021). Global, regional, and national prevalence, disability adjusted life years, and time trends for refraction disorders, 1990–2019: findings from the global burden of disease study 2019. BMC Public Health.

[13] Han X, Zou M, Liu Z, Sun Y, Young CA, Zheng D et al. Global burden of Cataract and its Association with Socioeconomic Development Status, 1990–2019. Available at SSRN 3869655. 2021.

[14] Yang X, Chen H, Zhang T, Yin X, Man J, He Q et al. Global, regional, and national burden of blindness and vision loss due to common eye diseases along with its attributable risk factors from 1990 to 2019: a systematic analysis from the global burden of disease study 2019. Aging.13(15):19614–42. 10.18632/aging.203374 PMC8386528 34371482

[15] Safi S, Ahmadieh H, Katibeh M, Yaseri M, Ramezani A, Shahraz S (2018). Burden of vision loss in the Eastern Mediterranean region, 1990–2015: findings from the global burden of Disease 2015 study. Int J Public Health.

[16] Kahloun R, Khairallah M, Resnikoff S, Cicinelli MV, Flaxman SR, Das A (2019). Prevalence and causes of vision loss in North Africa and Middle East in 2015: magnitude, temporal trends and projections. Br J Ophthalmol.

[17] Vos T, Lim SS, Abbafati C, Abbas KM, Abbasi M, Abbasifard M (2020). Global burden of 369 diseases and injuries in 204 countries and territories, 1990–2019: a systematic analysis for the global burden of Disease Study 2019. The Lancet.

[18] Murray CJL, Aravkin AY, Zheng P, Abbafati C, Abbas KM, Abbasi-Kangevari M (2020). Global burden of 87 risk factors in 204 countries and territories, 1990–2019: a systematic analysis for the global burden of Disease Study 2019. The Lancet.

[19] Wang Y. Smoothing splines: methods and applications. Chapman and Hall/CRC; 2011.

[20] Zhang R-H, Liu Y-M, Dong L, Li H-Y, Li Y-F, Zhou W-D, et al. Prevalence, years lived with disability, and Time Trends for 16 causes of blindness and vision impairment: findings highlight retinopathy of Prematurity. Front Pead. 2022. 10. 10.3389/fped.2022.735335 PMC8962664 35359888

[21] Burton MJ, Ramke J, Marques AP, Bourne RRA, Congdon N, Jones I (2021). The Lancet Global Health Commission on Global Eye Health: vision beyond 2020. The Lancet Global Health.

[22] Khandekar R, Mohammed AJ, Raisi AA (2007). Prevalence and causes of blindness & low vision; before and five years after ‘VISION 2020’ initiatives in Oman: a review. Ophthalmic Epidemiol.

[23] Damari B, Mahdavi A, Hajian M (2019). How to improve Iranians’ vision health: on the national policy of preventing Iranians’ blindness. Int J Ophthalmol.

[24] Ulldemolins AR, Lansingh VC, Valencia LG, Carter MJ, Eckert KA (2012). Social inequalities in blindness and visual impairment: a review of social determinants. Indian J Ophthalmol.

[25] Owsley C (2011). Aging and vision. Vision Res.

[26] Boyers LN, Karimkhani C, Hilton J, Richheimer W, Dellavalle RP (2015). Global burden of Eye and Vision Disease as reflected in the Cochrane database of systematic reviews. JAMA Ophthalmol.

[27] Lee CM, Afshari NA (2017). The global state of cataract blindness. Curr Opin Ophthalmol.

[28] Badr IA (1993). The scope of the cataract problem in the Middle East and the Mediterranean. Int Ophthalmol.

[29] Wang Y, Lou L, Cao J, Shao J, Ye J (2020). Socio-economic disparity in global burden of near vision loss: an analysis for 2017 with time trends since 1990. Acta Ophthalmol.

