# Supplementary material for: The burden of vision loss in the Middle East and North Africa region, 1990–2019
Source: Arch Public Health. 2023 Sep 26;81:172. doi: 10.1186/s13690-023-01188-y (PMC10521494; doi:10.1186/s13690-023-01188-y)
Supplement: Supplementary file 1 — Additional File 1: Table S1. Sequelae for vision loss and the associated disability weights from the Global Burden of Disease 2019 Study. [file 13690_2023_1188_MOESM1_ESM.docx]

| **Table S1: Sequelae for vision loss and the associated disability weights from the Global Burden of Disease 2019 Study** | | |
| --- | --- | --- |
| **Health state**  **name** | **Health state description** | **Disability weight**  **(95% CI)** |
| Near Vision Loss | This person has difficulty seeing things that are closer than 3 feet, if uncorrected by reading glasses, but has no difficulty with seeing things at a distance. | 0.011  (0.005–0.02) |
| Distance vision,  moderate loss | This person has vision problems that make it difficult to recognise faces or objects across a room. | 0.031  (0.019–0.049) |
| Distance vision,  severe loss | This person has severe vision loss, which causes difficulty in daily activities, some emotional impact (for example, worry), and some difficulty going outside the home without assistance. | 0.184  (0.125–0.259) |
| Distance vision,  blindness | This person is completely blind, which causes great difficulty in some daily activities, worry and anxiety, and great difficulty going outside the home without assistance. | 0.187  (0.124–0.26) |

Abbreviations: CI: Confidence interval.
