# Supplementary material for: The burden of vision loss in the Middle East and North Africa region, 1990–2019
Source: Arch Public Health. 2023 Sep 26;81:172. doi: 10.1186/s13690-023-01188-y (PMC10521494; doi:10.1186/s13690-023-01188-y)
Supplement: Supplementary file 2 — Additional File 2: Table S2. Prevalence of vision loss in 1990 and 2019 for both sexes and the percentage change in the age-standardised rates (ASRs) per 100,000 in the North Africa and the Middle East region. (Generated from data available from http://ghdx.healthdata.org/gbd-results-tool). [file 13690_2023_1188_MOESM2_ESM.docx]

| **Table S2: Prevalence of vision loss in 1990 and 2019 and the percentage change in the age-standardised rates (ASRs) per 100,000 in the North Africa and the Middle East region**  **(Generated from data available from http://ghdx.healthdata.org/gbd-results-tool)** | | | | | |
| --- | --- | --- | --- | --- | --- |
|  | **1990** | | **2019** | | **Percentage change in ASRs per 100,000** |
|  | **No (95% UI)** | **ASRs per 100,000 (95% UI)** | **No (95% UI)** | **ASRs per 100,000 (95% UI)** |  |
| **North Africa and Middle East** | **15982219 (14187331 , 17842726)** | **7915.2 (6993.7 , 8953.6)** | **32493045 (28615366 , 36578053)** | **7040 (6195 , 8002.7)** | **-11.1 (-12.5 , -9.7)** |
| **Afghanistan** | **694376 (610407 , 785621)** | **8994.2 (7925.3 , 10203.6)** | **1474739 (1314180 , 1645083)** | **8669.4 (7701.8 , 9803.8)** | **-3.6 (-7.7 , 0.4)** |
| **Algeria** | **1183420 (1045387 , 1329565)** | **8094.4 (7120.5 , 9179.5)** | **2511456 (2197306 , 2862411)** | **7245 (6385.7 , 8220.5)** | **-10.5 (-13.8 , -6.5)** |
| **Bahrain** | **19252 (17021 , 21660)** | **7749.2 (6821.1 , 8722.3)** | **76991 (66529 , 89429)** | **6935.5 (6090.8 , 7906.9)** | **-10.5 (-14 , -7.4)** |
| **Egypt** | **2890941 (2571073 , 3229007)** | **8527.3 (7551.9 , 9553.7)** | **5286491 (4663456 , 5958968)** | **7587.6 (6689.1 , 8589)** | **-11 (-14.9 , -7.6)** |
| **Iran** | **2672171 (2410092 , 2947047)** | **8123.6 (7345.9 , 8974.6)** | **5189421 (4674982 , 5752587)** | **7008.8 (6299.5 , 7778)** | **-13.7 (-14.7 , -12.8)** |
| **Iraq** | **769473 (681521 , 865811)** | **8148 (7168 , 9263.1)** | **1894229 (1668248 , 2145094)** | **7188.6 (6332 , 8194.2)** | **-11.8 (-15.5 , -8)** |
| **Jordan** | **122389 (107127 , 138529)** | **7071.5 (6115.3 , 8067.5)** | **466915 (405676 , 535643)** | **6310.5 (5472.6 , 7295.9)** | **-10.8 (-14.8 , -7.2)** |
| **Kuwait** | **62076 (54514 , 69539)** | **7274.8 (6396.8 , 8218.5)** | **201027 (175623 , 228226)** | **6651.8 (5863.9 , 7604.1)** | **-8.6 (-12.2 , -4.9)** |
| **Lebanon** | **207405 (184777 , 229776)** | **8427.8 (7481.2 , 9380.3)** | **385543 (342104 , 435490)** | **7436.8 (6593 , 8386.3)** | **-11.8 (-15.8 , -7.7)** |
| **Libya** | **178954 (158904 , 200580)** | **7838.8 (6896.8 , 8868.3)** | **376092 (329144 , 427264)** | **7050.9 (6199.1 , 8055.7)** | **-10.1 (-13.7 , -6.7)** |
| **Morocco** | **1165359 (1022152 , 1335180)** | **7546 (6576 , 8724.4)** | **2184191 (1873215 , 2523626)** | **6959.9 (6023.5 , 8005.7)** | **-7.8 (-11.9 , -3.1)** |
| **Oman** | **93010 (82954 , 103871)** | **8673.2 (7634.2 , 9898.6)** | **241576 (214912 , 267605)** | **9139 (8275.2 , 10048.7)** | **5.4 (-0.6 , 12.5)** |
| **Palestine** | **89023 (78691 , 100430)** | **8302.1 (7274.4 , 9439)** | **204793 (180608 , 231746)** | **7198.3 (6302.3 , 8226.7)** | **-13.3 (-16.7 , -9.3)** |
| **Qatar** | **14943 (13061 , 16961)** | **7789.6 (6888.3 , 8781.7)** | **102405 (88628 , 117818)** | **6790.4 (5968.2 , 7648.3)** | **-12.8 (-16.2 , -9.3)** |
| **Saudi Arabia** | **866643 (787898 , 947354)** | **10548.6 (9625.2 , 11542.3)** | **1824520 (1641659 , 2026299)** | **8343.9 (7539.1 , 9197.3)** | **-20.9 (-23.8 , -18)** |
| **Sudan** | **934471 (829368 , 1045763)** | **8536.4 (7539.3 , 9668.9)** | **1660823 (1463490 , 1863433)** | **7423.2 (6473.5 , 8474.1)** | **-13 (-16.6 , -9.5)** |
| **Syrian Arab Republic** | **553679 (494392 , 618893)** | **8324.1 (7381.9 , 9409.6)** | **929351 (811162 , 1053224)** | **7392.1 (6532.1 , 8363)** | **-11.2 (-14.3 , -7.7)** |
| **Tunisia** | **400234 (349189 , 457279)** | **7329.1 (6384 , 8355.5)** | **791531 (673946 , 927901)** | **6454.9 (5518.5 , 7537.4)** | **-11.9 (-15.7 , -7.9)** |
| **Turkey** | **2533854 (2171344 , 2927112)** | **6499 (5555.5 , 7590.2)** | **5118215 (4339121 , 5961223)** | **5923.4 (5060.1 , 6903.4)** | **-8.9 (-14 , -3.7)** |
| **United Arab Emirates** | **57862 (50430 , 65545)** | **7599.8 (6693.8 , 8630.3)** | **356229 (301396 , 412926)** | **6690.6 (5880.4 , 7633.2)** | **-12 (-15.1 , -8.8)** |
| **Yemen** | **461935 (400520 , 535084)** | **7469.5 (6357.4 , 8787.3)** | **1183493 (1033235 , 1344694)** | **7385.7 (6405.5 , 8500.9)** | **-1.1 (-6.6 , 4.6)** |
