# Supplementary material for: The burden of vision loss in the Middle East and North Africa region, 1990–2019
Source: Arch Public Health. 2023 Sep 26;81:172. doi: 10.1186/s13690-023-01188-y (PMC10521494; doi:10.1186/s13690-023-01188-y)
Supplement: Supplementary file 3 — Additional File 3: Table S3. YLDs due to vision loss in 1990 and 2019 for both sexes and the percentage change in the age-standardised rates (ASRs) per 100,000 in the North Africa and the Middle East region. (Generated from data available from http://ghdx.healthdata.org/gbd-results-tool). [file 13690_2023_1188_MOESM3_ESM.docx]

| **Table S3: YLDs due to vision loss in 1990 and 2019 and the percentage change in the age-standardised rates (ASRs) per 100,000 in the North Africa and the Middle East region**  **(Generated from data available from http://ghdx.healthdata.org/gbd-results-tool)** | | | | | |
| --- | --- | --- | --- | --- | --- |
|  | **1990** | | **2019** | | **Percentage change in ASRs per 100,000** |
|  | **No (95% UI)** | **ASRs per 100,000 (95% UI)** | **No (95% UI)** | **ASRs per 100,000 (95% UI)** |  |
| **North Africa and Middle East** | **783862 (556168 , 1076910)** | **415.5 (296.1 , 557.4)** | **1392915 (974014 , 1910579)** | **314.5 (222.1 , 427.6)** | **-24.3 (-27.6 , -20.8)** |
| **Afghanistan** | **35057 (25049 , 47795)** | **485.1 (346.6 , 651.6)** | **70486 (49969 , 97331)** | **469.6 (333 , 632.8)** | **-3.2 (-8.2 , 2.4)** |
| **Algeria** | **57333 (40499 , 78520)** | **423.2 (301.3 , 570.6)** | **107179 (75493 , 146663)** | **321.4 (227.7 , 436.8)** | **-24.1 (-27.6 , -20)** |
| **Bahrain** | **855 (588 , 1175)** | **384.2 (274.2 , 516.5)** | **2871 (1974 , 4061)** | **286.9 (201.3 , 392.2)** | **-25.3 (-29.5 , -20.9)** |
| **Egypt** | **137652 (96514 , 189839)** | **441.1 (315 , 595.5)** | **216390 (148682 , 300116)** | **330.5 (230.6 , 453.4)** | **-25.1 (-28.9 , -21)** |
| **Iran** | **148263 (105176 , 200835)** | **500.5 (357.7 , 670)** | **270459 (191734 , 365108)** | **376.1 (267.1 , 505.8)** | **-24.9 (-27.2 , -22.2)** |
| **Iraq** | **36667 (26110 , 50166)** | **415.9 (296.2 , 558.1)** | **78123 (53982 , 108201)** | **316.4 (224 , 429.6)** | **-23.9 (-27.6 , -20)** |
| **Jordan** | **4948 (3412 , 6911)** | **311.5 (220.7 , 422.7)** | **16879 (11445 , 23944)** | **240.7 (167.7 , 334.3)** | **-22.7 (-26.8 , -18.5)** |
| **Kuwait** | **2571 (1767 , 3581)** | **335.7 (237.7 , 456)** | **7646 (5182 , 10814)** | **271.7 (191.1 , 373.8)** | **-19.1 (-22.4 , -15.5)** |
| **Lebanon** | **9671 (6818 , 13146)** | **415.3 (293.8 , 561.1)** | **15959 (11170 , 21721)** | **307.7 (215.6 , 419.2)** | **-25.9 (-30.2 , -20.9)** |
| **Libya** | **8895 (6349 , 12136)** | **423.4 (302.3 , 571.7)** | **16762 (11915 , 22769)** | **330.2 (233.9 , 448.1)** | **-22 (-26.9 , -16.8)** |
| **Morocco** | **49766 (35029 , 68147)** | **337.6 (239.7 , 455.3)** | **83195 (57658 , 116064)** | **275.4 (193 , 379.9)** | **-18.4 (-23 , -13.7)** |
| **Oman** | **4260 (2968 , 5900)** | **447.4 (317.8 , 603.4)** | **10623 (7367 , 14582)** | **455 (325.7 , 615.3)** | **1.7 (-6 , 9.7)** |
| **Palestine** | **4075 (2903 , 5632)** | **407 (291 , 551.6)** | **7957 (5452 , 11096)** | **299.4 (210.7 , 410.3)** | **-26.4 (-30.2 , -22.6)** |
| **Qatar** | **661 (459 , 919)** | **410 (292.2 , 549)** | **3840 (2602 , 5445)** | **287.4 (202.4 , 394.4)** | **-29.9 (-35.2 , -24.4)** |
| **Saudi Arabia** | **57065 (40710 , 77820)** | **803.6 (572.7 , 1077)** | **89963 (63397 , 122806)** | **464.8 (332 , 621.9)** | **-42.2 (-46.1 , -37.8)** |
| **Sudan** | **51897 (36872 , 70541)** | **509.9 (365.7 , 686.9)** | **77359 (54679 , 106068)** | **373.4 (266.5 , 503.1)** | **-26.8 (-31.4 , -21.3)** |
| **Syrian Arab Republic** | **27188 (19164 , 37309)** | **444.8 (315.2 , 595.3)** | **39642 (27816 , 54424)** | **331.2 (233.8 , 451)** | **-25.6 (-29.3 , -21.3)** |
| **Tunisia** | **20351 (14431 , 27609)** | **397.5 (281.4 , 538.9)** | **33503 (23412 , 45643)** | **278.7 (196.6 , 378)** | **-29.9 (-34.4 , -25)** |
| **Turkey** | **103580 (72157 , 141750)** | **274.5 (192.7 , 371.5)** | **179407 (123563 , 248271)** | **210.7 (145.3 , 290.9)** | **-23.2 (-28.3 , -17.7)** |
| **United Arab Emirates** | **2487 (1703 , 3435)** | **380.3 (270.1 , 513)** | **13430 (9159 , 18934)** | **288.5 (202.2 , 392.1)** | **-24.1 (-27.5 , -20.5)** |
| **Yemen** | **20094 (14081 , 27819)** | **357.9 (255.4 , 482.3)** | **49827 (34834 , 68627)** | **335.5 (238 , 452.9)** | **-6.2 (-12 , 0.3)** |
