# Supplementary figures and images for: The burden of vision loss in the Middle East and North Africa region, 1990–2019
Source: Arch Public Health. 2023 Sep 26;81:172. doi: 10.1186/s13690-023-01188-y (PMC10521494; doi:10.1186/s13690-023-01188-y)

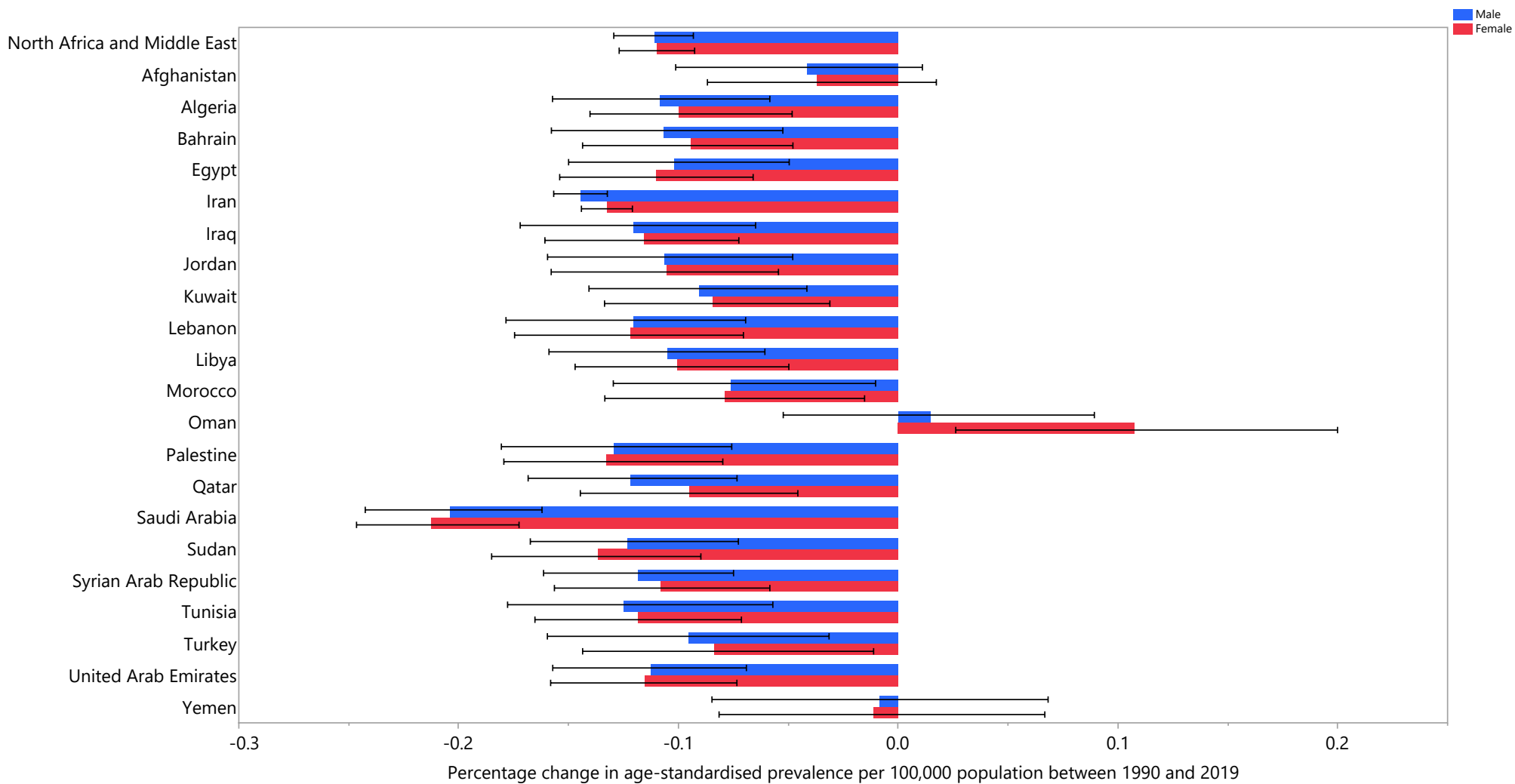

Supplement: Supplementary file 4 — Additional File 4: Figure S1. The percentage change in the age-standardised point prevalence of vision loss in the Middle East and North Africa region from 1990 to 2019, by sex and country. (Generated from data available from http://ghdx.healthdata.org/gbd-results-tool) [file 13690_2023_1188_MOESM4_ESM.pdf]

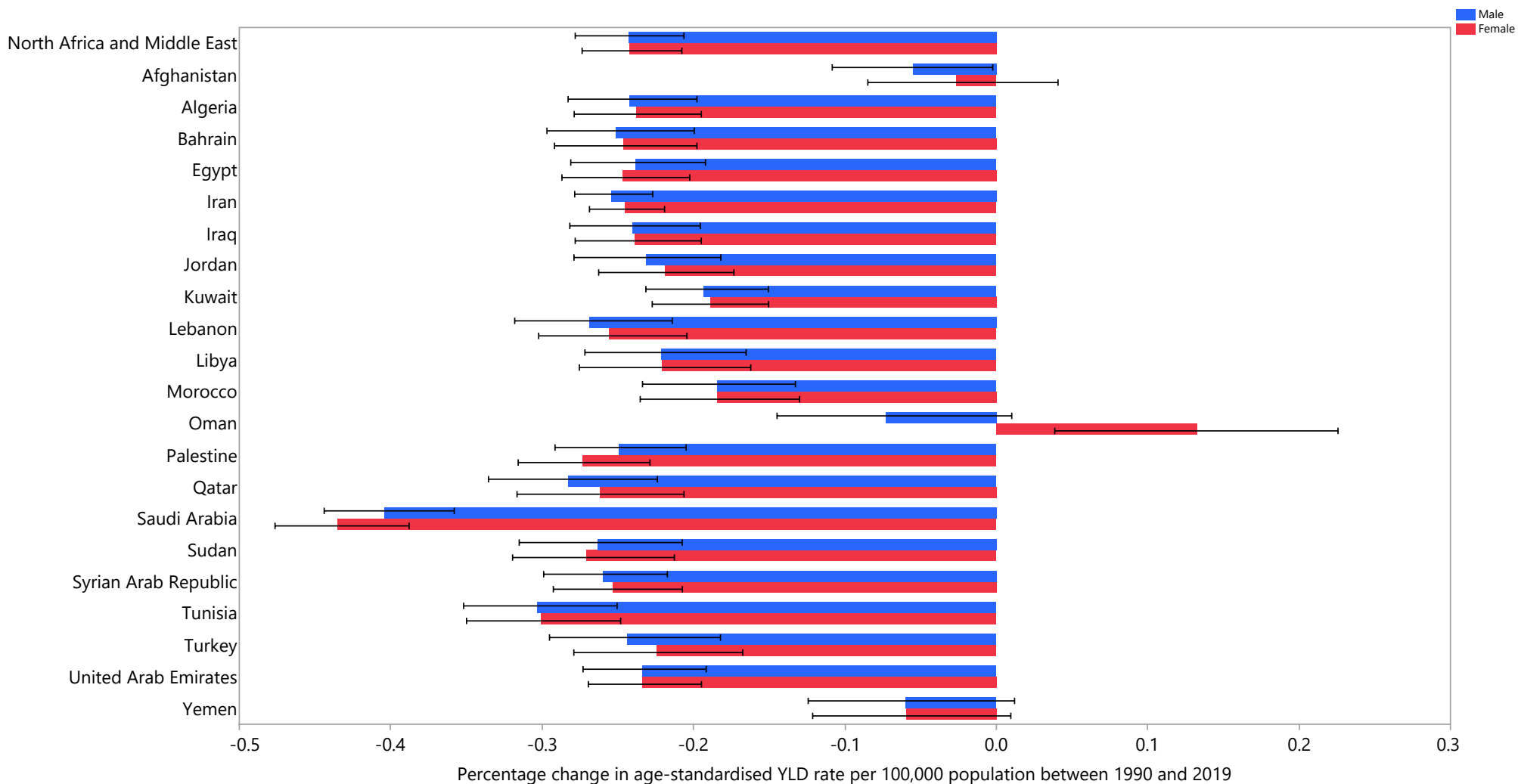

Supplement: Supplementary file 5 — Additional File 5: Figure S2. The percentage change in the age-standardised YLD rate of vision loss in the Middle East and North Africa region from 1990 to 2019, by sex and country. (Generated from data available from http://ghdx.healthdata.org/gbd-results-tool) [file 13690_2023_1188_MOESM5_ESM.pdf]
